# Alternate Day Fasting Combined with a Low Carbohydrate Diet: Effect on Sleep Quality, Duration, Insomnia Severity and Risk of Obstructive Sleep Apnea in Adults with Obesity

**DOI:** 10.3390/nu13010211

**Published:** 2021-01-13

**Authors:** Faiza Kalam, Kelsey Gabel, Sofia Cienfuegos, Mark Ezpeleta, Eric Wiseman, Krista A. Varady

**Affiliations:** Department of Kinesiology and Nutrition, University of Illinois at Chicago, Chicago, IL 60612, USA; fkalam2@uic.edu (F.K.); kdipma2@uic.edu (K.G.); scienf2@uic.edu (S.C.); mezpel2@uic.edu (M.E.); ewisem2@uic.edu (E.W.)

**Keywords:** intermittent fasting, alternate day fasting, low carbohydrate diet, sleep quality, insomnia, obstructive sleep apnea, obesity, weight loss

## Abstract

**Background**: Alternate day fasting combined with a low carbohydrate diet (ADF-LC) is an effective weight loss regimen. Whether the weight loss induced by ADF-LC can improve sleep, remains unknown. Objective: This study examined the effect an ADF-LC diet on sleep quality, duration, insomnia severity and the risk of obstructive sleep apnea. **Methods**: Adults with obesity (*n* = 31) participated in ADF (600 kcal “fast day”; ad libitum intake “feast day”) with a low-carbohydrate diet (30% carbohydrates, 35% protein, and 35% fat). The 6-month trial consisted of a 3-month weight loss period followed by a 3-month weight maintenance period. **Results**: Reductions in body weight (−5 ± 1 kg, *p* < 0.001) and fat mass (−4 ± 1 kg, *p* < 0.01) were noted during the weight loss period, and these reductions were sustained during the weight maintenance period. Lean mass and visceral fat remained unchanged. The Pittsburgh Sleep Quality Index (PSQI) score indicated poor sleep quality at baseline (6.4 ± 0.7) with no change by month 3 or 6, versus baseline. ISI score indicated subthreshold insomnia at baseline (8.5 ± 1.0), with no change by month 3 or 6, versus baseline. The percent of subjects with high risk of obstructive sleep apnea at baseline was 45%, with no change by month 3 or 6. Wake time, bedtime, and sleep duration remained unchanged. **Conclusion**: The ADF-LC diet does not impact sleep quality, duration, insomnia severity or the risk of obstructive sleep apnea in adults with obesity.

## 1. Introduction

The prevalence of obesity in the United States is 42% [1]. Obesity is associated with short sleep duration (<7 h/night) and poor sleep quality [2,3,4,5,6]. In addition, the prevalence of obstructive sleep apnea is higher in subjects with obesity [7,8]. Weight loss by dietary restriction may improve sleep quality by reducing sleep fragmentation and alleviating sleep-disordered breathing [9,10]. On the other hand, good sleep quality and longer sleep duration may promote weight loss by reducing appetite [11] and decreasing the window of time an individual has to consume extra calories [12,13]. These findings highlight the delicate interplay that exists between sleep and body weight.

Alternate day fasting (ADF) has gained popularity as a weight loss regimen in recent years. ADF consists of a “feast day” where food is consumed ad libitum over 24 h, alternated with a “fast day” where intake is limited to approximately 600 kcal over 24 h. Human trials report consistent reductions in body weight (3–7%) and improvements in metabolic parameters after 2–12 months of ADF [14,15,16]. However, no study to date has examined whether the weight loss induced by ADF can improve sleep in adults with obesity.

Another factor that influences sleep is the macronutrient composition of the diet. A few studies have examined how manipulating carbohydrate and protein intake effect sleep variables. Afaghi et al. [17] demonstrated that a very low carbohydrate diet (10% of energy) increased the percentage of deep sleep episodes compared to a control mixed diet. In contrast, epidemiological evidence suggests that low carbohydrate diets (10–20% of energy) are associated with difficulty maintaining sleep [18]. As for the effects of protein intake on sleep, a recent study showed that a high protein diet (55% of energy) was associated with fewer wake episodes [19]. These findings are complementary to the those of Zhou et al. [20], which demonstrate that high protein diets (>25% of energy) improve global sleep quality in individuals with overweight. In contrast, Castro et al. [21] reported no significant difference in sleep quality after four months of a high protein diet. Thus, the effects of carbohydrate and protein intake on sleep remain unclear.

We recently conducted a clinical trial to examine how ADF combined with a low carbohydrate diet (ADF-LC) effects body weight and metabolic disease risk factors in adults with obesity [22]. Our findings show that the ADF-LC diet produced clinically significant reductions in body weight (6% from baseline), LDL cholesterol, blood pressure and fasting insulin levels [22]. The present study was conducted as a secondary analysis to examine how an ADF-LC diet impacts sleep. We hypothesized that six months of an ADF-LC diet would improve sleep quality, duration, insomnia severity, and risk of obstructive sleep apnea, and that these improvements would be more pronounced in subjects who were poor sleepers at baseline.

## 2. Materials and Methods

### 2.1. Subject Selection

Subjects were recruited from the Chicago land area by flyers. The inclusion criteria were as follows: body mass index (BMI) between 30–50 kg/m^2^; age 18–65 years; pre-menopausal or postmenopausal; lightly active for 3 months prior to the study; weight stable for 3 months prior to the beginning of the study (less than 4 kg weight loss or weight gain); nondiabetic; no history of cardiovascular disease; nonsmoker; not a night shift worker; not taking weight loss medications; and not planning to engage in trans-meridian travel 4-weeks prior to each sleep outcome assessment. The University of Illinois Chicago Office for the Protection of Research Subjects approved the experimental protocol (IRB approval #2017-1363), and all research participants gave their written informed consent to participate in the trial.

### 2.2. Alternate Day Fasting-Low Carbohydrate (ADF-LC) Diet Protocol

The trial consisted of a 1-month baseline period followed by a 6-month ADF-LC intervention period. During the baseline period, subjects continued their regular diet and exercise routines and were asked to remain weight stable. As such, each subject acted as their own control. The 6-month ADF-LC intervention period was divided into a 3-month weight-loss period followed by a 3-month weight maintenance period. The ADF intervention consisted of feast day alternated with a fast day. The macronutrient composition of the diet was as follows: 30% carbohydrates, 35% protein, and 35% fat. Participants were provided with low carbohydrate meal replacement shakes during both the weight loss and weight maintenance periods (each packet: 200 kcal, 10 g carbohydrates, 26 g protein, 6 g fat; Optifast HP Shake Mix, Nestle, Bridgewater, NJ, USA). The fast/feast days began at midnight each day.

#### 2.2.1. Weight Loss Period (Month 0–3)

On the fast day during the weight loss period, participants consumed 3 meal replacements per day (providing a total of 600 kcal). Subjects were not permitted to consume anything else on the fast day except for water, black coffee, and tea (without milk or sugar). On alternating feast days, subjects consumed 5 meal replacements that provided a total of 1000 kcal. After all the shakes were consumed on the feast day, participants were permitted ad libitum intake of low carbohydrate/high protein foods. Participants received dietary counseling from the study dietician to learn how to select low carbohydrate/high protein foods on the feast day to comply with their diet prescription.

#### 2.2.2. Weight Maintenance Period (Month 3–6)

On the fast day during the weight maintenance period, subjects continued to consume 3 meal replacements per day (providing 600 kcal). Subjects were not permitted to consume anything else on the fast day except for water, black coffee, and tea (without milk or sugar). On alternating feast days during the maintenance period, subjects consumed 3 meal replacements that provided a total of 600 kcal. After all the shakes were consumed on the feast day, participants were permitted ad libitum intake of low carbohydrate/high protein foods.

### 2.3. Body Weight, Body Composition, Physical Activity, and Diet Adherence

Body weight was assessed at the research center using a digital scale (Omron HBF-500; Omron HealthCare, Kyoto, Japan) at baseline, in month 3 and in month 6. Body composition (fat mass, lean mass, visceral fat mass) was measured using dual X-ray absorptiometry (iDXA, General Electric Inc., Chicago, IL, USA). Physical activity was assessed over a 7-day period at baseline, in month 3, and in month 6 by a pedometer (Yamax Digi-walker SW-200, Yamax Inc., Tokyo, Japan). Each subject was asked to maintain their level of physical activity during the 6-month trial so that it would not confound the results. Adherence with the intervention (i.e., meal replacement protocol) was assessed using a daily “Shake adherence log”. The logs were collected every two weeks. Percent adherence was calculated separately for the fast day and feast day as: % Adherence = [# shakes consumed/# of shakes distributed] × 100.

### 2.4. Sleep Measures

All sleep questionnaires were administered at baseline (prior to the intervention), in month 3 (the end of the weight loss period), and in month 6 (the end of the weight maintenance period). The Pittsburgh Sleep Quality Index (PSQI) [23] was used to measure sleep quality, timing and duration. The PSQI is a 19-item self-report and assesses total sleep quality in the past month, with a total score of 0–21. A total score >5 indicates poor sleep quality. The PSQI also assesses usual bedtime, usual wake time, and hours of actual obtained sleep. The Insomnia Severity Index (ISI) [24] was used to measure the severity of insomnia in the past week (7-item questionnaire, with a total score of 0–28). The total score for the ISI is interpreted as follows: no clinically significant insomnia (0–7), sub-threshold insomnia (8–14), moderate severity insomnia (15–21), and severe insomnia (22–28). The Berlin Questionnaire [25] was used to estimate the proportion of subjects at high risk for obstructive sleep apnea.

### 2.5. Statistical Analysis

All data are presented as means ± SEM. A repeated measures ANOVA was used to assess differences in outcome measures over time (baseline, month 3, and month 6). Post-hoc analyses were performed using the Bonferroni correction. McNemar’s test was used to evaluate changes in the proportion of subjects at risk for obstructive sleep apnea at months 3 and 6, versus baseline. The differences were considered significant at *p* < 0.05. All data were analyzed using SPSS software (version 27.0, SPSS Inc., Chicago, IL, USA).

A sub-analysis was also performed to examine the effects of the ADF-LC intervention in “good sleepers” and “poor sleepers”. Good sleepers were subjects with a PSQI total scores equal to or below 5 at baseline, while “poor sleepers” were subjects with a PSQI total score of greater than 5 at baseline. For this sub-analysis, a repeated measures ANOVA was used to assess the differences in outcome measures over time (baseline, month 3, and month 6) in “good sleepers” and in “poor sleepers”. Post-hoc analyses were performed using the Bonferroni correction.

## 3. Results

### 3.1. Body Weight, Body Composition, Physical Activity, and Diet Adherence

A total of 94 participants were screened, 52 were enrolled, and 31 completed the full 6-month protocol. On average, completers were middle-aged females with obesity (Table 1). Changes in body weight, body composition and physical activity over the 6-month trial are displayed in Table 1. In “all subjects”, body weight decreased (*p* < 0.001) by −5 ± 1 kg by month 3 (the end of the weight loss period), and by 6 ± 1 kg by month 6 (the end of the weight maintenance period), versus baseline. In “good sleepers”, body weight was reduced (*p* < 0.001) by −6 ± 2 kg by month 3, and by −6 ± 2 kg by month 6, relative to baseline. In “poor sleepers”, body weight decreased (*p* < 0.001) by −4 ± 2 kg by month 3, and by −6 ± 2 kg by month 6, compared to baseline. There were no statistically significant changes in body weight from month 3 to 6 in any group, indicating weight stability during the weight maintenance period. 

Fat mass was reduced (*p* < 0.01) at month 3 and 6 of the trial, relative to baseline, in all groups. Lean mass decreased (*p* < 0.01) by month 3, but was not statistically different by month 6, relative to baseline, in all groups. Visceral fat mass and physical activity (measured as steps/day) remained unchanged throughout the 6-month study in all groups. As for diet adherence, participants consumed 89% of the shakes prescribed on the fast days and 85% of the shakes prescribed on the feast days. This level of compliance with the meal replacement protocol remained stable (*p* = 0.72) over the course of the 6-month trial.

### 3.2. Sleep Quality

**Figure 1:**
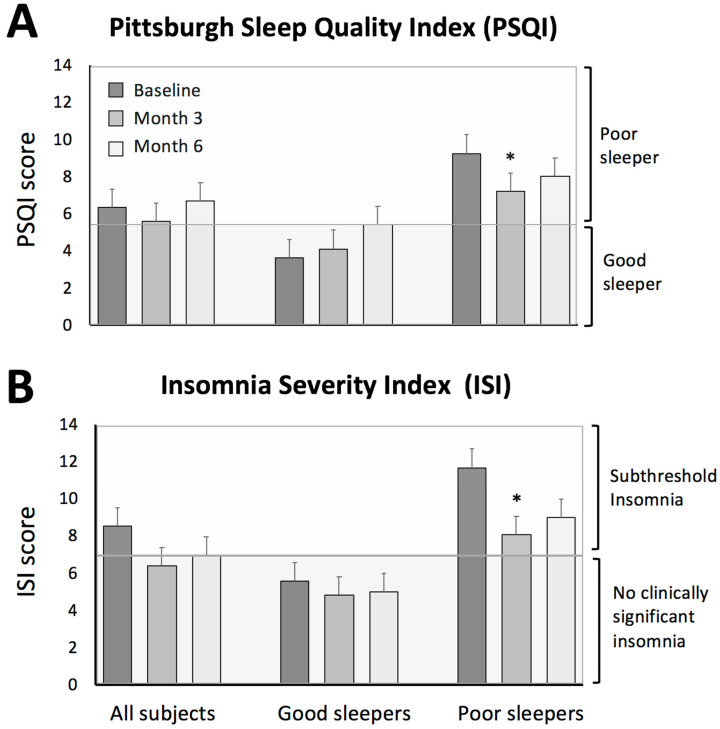
Sleep quality and insomnia severity during 6 months of alternate day fasting with a low carbohydrate diet. The values are reported as mean ± SEM. (**A**) Changes in sleep quality were evaluated by the Pittsburgh Sleep Quality Index (PSQI) questionnaire. “All subjects” represents the group of subjects as a whole (*n* = 31). Subjects were then divided into groups based on baseline sleep quality: “good sleepers” (*n* = 16) are subjects with a PSQI total score equal to or below 5 at baseline and “poor sleepers” are subjects with a PSQI total score greater than 5 at baseline (*n* = 15). (**B**) Changes in insomnia severity were evaluated by the Insomnia Severity Index (ISI). The ISI score is interpreted as follows: no clinically significant insomnia (0–7), subthreshold insomnia (8–14). * Significantly different from baseline within each group (ANOVA). No significant differences between months 3 and 6 for any parameter.

The PSQI was used to evaluate the quality, timing, and duration of sleep (Table 1 and Figure 1). The PSQI in “all subjects” was 6.4 ± 0.7 at baseline, indicating poor sleep quality. The PSQI score in “all subjects” did not change significantly by month 3 (the end of the weight loss period) or month 6 (the end of the weight maintenance period), versus baseline. In “good sleepers”, mean PSQI score was 3.6 ± 0.4, and did not change significantly by month 3 or 6, relative to baseline. In “poor sleepers”, the mean PSQI score significantly decreased (*p* < 0.05) from baseline (9.3 ± 0.9) to month 3 (7.2 ± 0.7). However, the PSQI score by month 6 (8.0 ± 0.9) was not significantly different versus baseline or month 3. Finally, wake time, bedtime, and sleep duration did not change at month 3 or 6, compared to baseline, in any group.

### 3.3. Insomnia Severity

The ISI survey was used to measure changes in insomnia severity (Table 1 and Figure 1). In “all subjects”, the ISI score indicated subthreshold insomnia at baseline (8.5 ± 1.0), with no statistically significant differences by month 3 or 6, versus baseline. In “good sleepers”, the ISI score indicated an absence of clinically significant insomnia at baseline (5.6 ± 0.9), and this did not change by month 3 or 6. In “poor sleepers”, the ISI score indicated subthreshold insomnia at baseline (11.7 ± 1.3). The ISI score in this group was reduced (*p* < 0.05) to 8.1 ± 1.4 by month 3 (the end of the weight loss period). However, by the end of the trial, the ISI score was no longer statistically significant versus baseline or month 3.

### 3.4. Risk of Obstructive Sleep Apnea

The risk for obstructive sleep apnea was present in 45% of “all subjects”, 38% of “good sleepers”, and 53% of “poor sleepers” at baseline (Table 1). There were no statistically significant changes in the risk for obstructive sleep apnea by month 3 or 6, relative to baseline, in any group.

## 4. Discussion

This study is the first to examine the effects of an ADF-LC diet on sleep in adults with obesity. Our findings show that this regimen produced clinically significant weight loss (6% from baseline) but had no effect on sleep quality, duration, insomnia severity, or risk for obstructive sleep apnea. We also performed a sub-analysis to examine whether poor sleepers would observe greater improvements in sleep with the ADF-LC diet. At the end of the weight loss period (month 3), poor sleepers experienced improvements in sleep quality accompanied by reductions in insomnia severity, versus baseline. However, these improvements were no longer significant by the end of the weight maintenance period (month 6).

The main goal of this study was to examine whether ADF combined with a low carbohydrate diet could improve sleep quality and insomnia severity in adults with obesity. Our findings show that neither sleep quality nor insomnia severity was improved by this diet. These results are in accordance with other recent studies that examine the effect of intermittent fasting on sleep [26,27,28]. For instance, in the study by Gabel et al. [26], subjects with obesity confined their eating window to 8-h per day. After 3 months of this 8-h time restricted eating (TRE) regimen, body weight decreased by 3% but sleep quality and insomnia severity remained unchanged. Similarly, Cienfuegos et al. [27] reported no change in sleep quality or insomnia severity with 4-h TRE or 6-h TRE after 2 months, despite 3% weight loss. Likewise, Wilkinson et al. [28] also reported no change in sleep quality after 2 months of 10-h TRE, despite 4% weight loss.

In our sub-analysis of “good” versus “poor” sleepers, we noted minor improvements in sleep quality and insomnia severity during the weight loss period, but these effects were washed out during the maintenance period. The reason why this occurred is uncertain, considering that the body weight reductions achieved during weight loss period (6%) were sustained during the maintenance period. Recent trials show that maintenance of weight loss is associated with lasting improvements in sleep quality and insomnia severity [13,29]. Nonetheless, it is likely that our study was not powered to identify significant changes in these secondary outcome measures of sleep. A well powered randomized controlled trial, which specifically examines how ADF-LC impacts sleep, will be needed before solid conclusions can be reached.

Our study also tested the effect of carbohydrate restriction on sleep. While our trial did not include a high carbohydrate ADF control group, we cannot rule out that the improvements in sleep may be partly mediated by the low carbohydrate diet. Several studies have examined the effects of manipulating macronutrient intake on sleep quality and duration. High carbohydrate diets may lead to longer sleep [30], and shorter wake time [31], while very low carbohydrate diets extend deep sleep [17]. This also parallels findings from an observational study, which reported that patients with type 2 diabetes who consumed fewer carbohydrates had better sleep status [32]. In addition, the high protein intake in our study (35% of energy) may have also impacted sleep. Lindseth et al. [19] reported significantly fewer wake episodes following a high protein (55% of energy) isocaloric diet over 4 days. Furthermore, poor sleepers with overweight or obesity may become good sleepers when following higher protein (30% of energy) energy-restricted diets [33]. In view of these findings, it is possible that the low carbohydrate/high protein diet implemented here may have contributed to the improvements in sleep noted in poor sleepers during the weight loss phase.

We did not observe any changes in sleep duration or timing during the ADF-LC intervention. Likewise, no effect on sleep duration and timing was observed in good versus poor sleepers in our sub-analysis. However, it should be noted that our participants had a mean sleep duration of ~7 h/night at baseline, which is close to the recommended 7 h minimum stipulated by the National Sleep Foundation [34]. Evidence suggests that 7–8 h of sleep per night is associated with better health and lower body weight [34]. Conversely, short sleep duration, poor sleep quality, and later bedtimes are associated with higher food consumption, poor diet, and obesity [35]. Thus, since our subjects were already getting sufficient hours of sleep, this could explain why no additional benefit was noted.

The risk of obstructive sleep apnea did not change significantly during the trial in any group. At baseline, close to half of our cohort (45%) was at high risk of obstructive sleep apnea. Although the percentage of subjects at risk for sleep apnea decreased numerically throughout the trial in each group, these changes were not statistically significant, relative to baseline. Thus, intermittent fasting in combination with carbohydrate restriction may not decrease the risk of obstructive sleep apnea in adults with obesity. These findings, however, will require further confirmation by studies that are designed to directly test this hypothesis.

Our study has several limitations. First, our study did not include a high carbohydrate/low protein control group. A randomized trial that includes this comparison group will be needed to truly assess the effect of this diet on sleep. This is major limitation to the study and severely impacts our ability to draw meaningful conclusions. Second, our sample size was small (*n* = 31). Since our power calculation was based exclusively on body weight, this study was likely not powered adequately to identify significant changes in these sleep parameters. Third, all measures of sleep were assessed via self-reported questionnaires. This study would have benefitted from the use of wrist actigraphy to provide more objective assessments of rest and activity patterns. Fourth, participants were allowed to consume caffeinated beverages. As such, certain subjects may have consumed caffeine late into the evening, which could have impacted their sleep quality and duration. Future ADF trials should control the timing of caffeine consumption to eliminate its effect on sleep quality and insomnia severity. Fifth, the majority of our sample was female (80%) which may have impacted the generalizability of our findings. Some studies suggest that obesity is associated with shorter sleep duration and greater insomnia symptoms in women but not in men [36,37]. As such, it will be important for future research to directly compare the effect of fasting on body weight and sleep in men versus women. Lastly, we did not assess the chronotype of our subjects at baseline by implementing the morningness-eveningness questionnaire [38]. Although it does not appear as though the participants were extreme chronotypes (based on reported sleep times), it is possible that there was some variability in individual chronotypes. The evening chronotype is associated with eating larger meals later in the day, and higher rates of sleep apnea in adults with obesity [39]. In view of this, it will be important to examine how the sleep habits of individuals of varying chronotypes respond to intermittent fasting.

In summary, these preliminary findings suggest that the ADF-LC diet does not impact sleep quality, duration, insomnia severity or the risk of obstructive sleep apnea in adults with obesity. Interestingly, this lack of effect occurred even though our subjects achieved clinically significant weight loss (6%) with the ADF-LC diet over six months. While our findings report no positive effects, it is important to note that this intermittent fasting regimen did not negatively impact sleep. For instance, sleep quality was not worsened, and sleep duration was not shortened. Thus, ADF combined with a low carbohydrate diet may be viewed as an effective weight loss therapy that does not detrimentally effect sleep in adults with obesity.

## Figures and Tables

**Table 1:** Body weight, body composition, and sleep variables during six months of alternate day fasting with a low carbohydrate diet.

	All Subjects (*n* = 31)			Good Sleepers (*n* = 16)			Poor Sleepers (*n* = 15)		
	Baseline	Month 3	Month 6	Baseline	Month 3	Month 6	Baseline	Month 3	Month 6
**Demographics**									
Age	48 ± 2			49 ± 3			48 ± 2		
Sex (Female/Male)	25/6			12/4			13/2		
Race/Ethnicity									
White	1			0			1		
Black	18			9			9		
Asian	3			3			0		
Hispanic	9			4			5		
**Anthropometrics**									
Body weight (kg)	100 ± 3	95 ± 3 *	94 ± 4 *	100 ± 5	94 ± 5 *	94 ± 5 *	100 ± 5	96 ± 5 *	94 ± 5 *
Fat mass (kg)	46 ± 2	42 ± 2 *	41 ± 3 *	46 ± 3	42 ± 3 *	41 ± 3 *	45 ± 3	42 ± 4 *	40 ± 4 *
Lean mass (kg)	49 ± 2	48 ± 1 *	49 ± 2	49 ± 2	48 ± 2 *	49 ± 2	50 ± 2	48 ± 2 *	**49 ± 2**
Visceral fat mass (kg)	1.4 ± 0.1	1.2 ± 0.1	1.2 ± 0.1	1.4 ± 0.2	1.3 ± 0.2	1.3 ± 0.2	1.3 ± 0.1	1.2 ± 0.2	1.2 ± 0.2
**Steps/day**	6931 ± 842	8070 ± 989	7648 ± 773	8104 ± 1365	8852 ± 1496	8449 ± 1168	6019 ± 1019	7461 ± 1391	7025 ± 992
**Pittsburgh Sleep Quality Index (PSQI)**									
Total score	6.4 ± 0.7	5.6 ± 0.5	6.7 ± 0.7	3.6 ± 0.4	4.1 ± 0.6	5.4 ± 1.1	9.3 ± 0.9	7.2 ± 0.7 *	8.0 ± 0.9
Wake time (h:min)	6:05 ± 0:25	6:20 ± 0:30	6:00 ± 0:20	5:50 ± 0:20	5:45 ± 0:20	5:40 ± 0:20	5:20 ± 0:15	5:35 ± 0:20	5:20 ± 0:25
Bedtime (h:min)	22:30 ± 0:20	22:15 ± 0:15	22:10 ± 0:25	22:20 ± 0:15	22:10 ± 0:20	22:00 ± 0:20	22:35 ± 0:15	22:20 ± 0:15	22:20 ± 0:20
Sleep duration (h:min)	7:35 ± 0:20	8:05 ± 0:25	7:50 ± 0:20	7:30 ± 0:15	7:35 ± 0:20	7:40 ± 0:20	6:45 ± 0:15	7:15 ± 0:20	7:00 ± 0:20
**Insomnia severity index (ISI)**									
Total score	8.5 ± 1.0	6.4 ± 0.9	6.9 ± 1.1	5.6 ± 0.9	4.8 ± 1.0	5.0 ± 1.6	11.7 ± 1.3	8.1 ± 1.4 *	9.0 ± 1.5
**Berlin questionnaire**									
High risk of obstructive sleep apnea (%)	45%	42%	32%	38%	38%	31%	53%	47%	33%

Values are reported as mean ± SEM. Good sleepers: subjects with a baseline total Pittsburgh Sleep Quality Index (PSQI) score ≤ 5. Poor sleepers: subjects with a baseline total Pittsburgh Sleep Quality Index (PSQI) score > 5. * (Bold) Significantly different from baseline within each group (ANOVA). No significant differences between months 3 and 6 for any parameter.

## Data Availability

The data presented in this study are available on request from the corresponding author.
